# Usefulness of Molecular Methods for *Helicobacter pylori* Detection in Pediatric Patients and Their Correlation with Histopathological Sydney Classification

**DOI:** 10.3390/ijms24010179

**Published:** 2022-12-22

**Authors:** Tomasz Bogiel, Agnieszka Mikucka, Anna Szaflarska-Popławska, Dariusz Grzanka

**Affiliations:** 1 Department of Microbiology, Ludwik Rydygier Collegium Medicum in Bydgoszcz, Nicolaus Copernicus University, 85-094 Bydgoszcz, Poland; 2 Clinical Microbiology Laboratory, Dr Antoni Jurasz University Hospital No. 1, 85-094 Bydgoszcz, Poland; 3 Department of Pediatric Endoscopy and Gastrointestinal Function Testing, Ludwik Rydygier Collegium Medicum in Bydgoszcz, Nicolaus Copernicus University, 85-094 Bydgoszcz, Poland; 4 Department of Clinical Pathomorphology, Faculty of Medicine, Collegium Medicum in Bydgoszcz, Nicolaus Copernicus University, 85-094 Bydgoszcz, Poland

**Keywords:** *Helicobacter pylori*, histopathological investigation, molecular diagnostics, PCR, pediatric patients, real-time PCR, Sydney modified classification

## Abstract

*Helicobacter pylori* infections, as one of the most prevalent among humans, are generally acquired during childhood, and are one of the main causes of chronic gastritis and peptic ulcer disease. A bacterial culture from a gastric biopsy is the gold standard and is the only method that has 100% specificity. However, its sensitivity varies, depending on experience of the laboratory staff, applied culture media, specimen transport conditions, biopsy site, and quality of the sample. The same factors compromise all invasive methods and a culture-based *H. pylori* infection diagnostic, as well as a recent intake of antibiotics, bismuth-containing compounds, and proton pump inhibitors. Molecular methods have been used for clinical microbiology investigation since the beginning of the 21st century. However, their usefulness for *H. pylori* infections diagnosis remains unclear, especially in pediatric patients. The aim of the study was to assess the incidence of *H. pylori* infections in a group of 104 pediatric patients and to compare the results of the PCR test with the corresponding histopathological investigation effects. Among the biopsy samples collected from 104 children, 44 (42.3%) were positive in PCR, while 43 (41.3%) and 39 (37.5%) presented histologically-confirmed signs of inflammation and *H. pylori* colonization, respectively. Moreover, the mean grades of the parameters of the histopathological examination were higher in the group of PCR-positive samples. The compatibility of both research methods was confirmed, emphasizing the usefulness of molecular methods for detecting *H. pylori* infections in pediatric patients. Considering that the PCR-based method gives reliable results and is less time-consuming and costly, it is worth discussing this method as a new standard in the diagnosis of *H. pylori* infections, at least among pediatric patients, for which culture-based diagnostics is not sufficient or histopathological examination is negative, while inflammation signs are observed macroscopically.

## 1. Introduction

*Helicobacter pylori* is a microaerophilic Gram-negative rod, one of the most prevalent human pathogens, generally acquired during childhood. In children, it is one of the main causes of chronic gastritis and peptic ulcer disease. It is also suspected to be related to refractory iron deficiency anemia and chronic immune thrombocytopenic purpura. The role of *H. pylori* infection in children’s growth or failure to thrive remains controversial [1].

*H. pylori* infections can be diagnosed by either invasive tests performed on biopsy samples obtained during upper gastrointestinal endoscopy (e.g., rapid urease test (RUT) histology, and culture) or by non-invasive methods (e.g., saliva antigen test, serological investigation, urea breath test, and stool antigen test) [2,3]. Polymerase chain reaction (PCR) can by categorized as both invasive and non-invasive test, depending on the specimen type used for the testing [4].

According to the current ESPGHAN/NASPGHAN guidelines, a diagnosis of *H. pylori* infection in children should be based on either a positive culture or both a positive histopathology and biopsy-based test (RUT or PCR) [5]. The lack of a single test that offers full sensitivity and specificity is a major disadvantage for the diagnosis of *H. pylori* infections [5].

According to some studies, the bacterial culture of gastric biopsy as a gold standard is the only method that has 100% specificity and it has the highest sensitivity (2.0 × 10 colony forming units (CFU) versus 2.0 × 10^2^ CFU in PCR-based method) [6]. Meanwhile, culture-based methods’ sensitivity results vary depending on the applied culture media, biopsy site, quality of the samples, etc. Furthermore, culture-based methods for *H. pylori* detection are compromised by the recent intake of drugs by patients, mostly antimicrobials and proton pump inhibitors (PPI) [7].

The histopathological examination provides a comprehensive assessment of the pathological changes associated with *H. pylori* infection, including the location of the gastritis, its type (chronic or acute), the presence of lymphoid follicles, intestinal metaplasia, gastric mucosa atrophy, and malignancy [4]. Therefore, the updated Sydney 0–3 graded classification is recommended for the standardization of a histopathological assessment of gastritis [8]. To achieve the highest possible diagnostic accuracy, it is recommended to collect gastric biopsy samples from multiple locations due to the diverse bacteria distribution within the mucous membranes [7].

In a few pediatric studies published to date, gastric biopsy-based PCR tests have been shown to be a reliable method for the detection of *H. pylori,* even at a low bacterial density, observed in minimally inflamed gastric mucosa or in PPI recipients [7,9]. Furthermore, PCR-based methods also allow for the detection of specific point mutations leading to resistance to some antimicrobials, e.g., clarithromycin and fluoroquinolones [9]. Further comparative studies are required to obtain evidence of the accuracy of these tests for a diagnosis of *H. pylori* infection in children. Thus, the aim of the study was to evaluate the usefulness of molecular-based methodology (through a commercially available test as well as “in house” *ureA* gene detection and sequencing of the DNA for the 16S rRNA-encoding gene for the verification of inconsistent results) for *H. pylori* detection in pediatric patients and to infer a possible correlation with the histopathological evaluation.

## 2. Results

*H. pylori* DNA was detected in 44 (42.3%) biopsy samples collected from 24 female and 20 male patients, with a median age of ± SD of 12 ± 4 years. The detailed results for each patient are presented in Supplementary Material Table S1.

The C_T_ values of the first PCR investigation for positive samples were in the range of 17.19–33.20 (mean ± SD reached 23.46 ± 4.03), and are given in Supplementary Material Table S1.

When checking the reproducibility of the PCR results, each individual DNA sample was subjected to PCR two or three times, and the re-testing results for each sample were the same as the initial ones. Therefore, the results were consistent each time and the study was repeatable. To avoid the expression of C_T_ values as the median C_T_, only the corresponding C_T_ values of the first PCR investigation for all of the patients are given in Supplementary Material Table S1.

A summary comparison of the results obtained with the application of PCR, with respect to the results of their histological investigation, is presented in Table 1, while the detailed evaluation of the results is presented in Supplementary Material, Tables S1 and S2.

There was a statistically significant difference between the applied method and the positive result that was obtained (*p* < 0.001, χ^2^ = 0.74372); the PCR method produced positive results statistically more often. Moreover, the sensitivity of the PCR-based methodology reached 95.3%, while the results of the specificity were 92.6% when compared with the histopathology results.

The distribution of the histopathological investigation grades noted in the group of 60 samples derived from 34 female and 26 male patients (median age ± SD; 12.5 ± 3.5 years) with negative PCR results is shown in Table 2, while the detailed results of the investigation are shown in Table S2, Supplementary Material. None of the patients included in this group presented visually recognizable symptoms typical for positive grade results (neutrophilic infiltrates, glandular atrophy, or intestinal metaplasia changes).

Signs of inflammation were noted among 40 (66.7%) patients in this group. *H. pylori* presence, confirmed with the histological evaluation, was detected in 2 (3.3%) out of 60 biopsy samples included in the group of patients with negative PCR results. Within this group, all of the observed histopathological changes with respect to *H. pylori* colonization density grading were assigned to the first grade in the modified Sydney scale, Supplementary Material Table S2.

One of the PCR-negative samples (number 64, Supplementary Material, Table S2) was positive for both the RUT and histology investigation (assigned to grade 1).

The histopathological investigation grade analysis revealed that in a group of PCR-negative samples, the highest value (0.8) was reached for the evaluation of the histologically assessed inflammation grade, as shown in Supplementary Material Table S2.

The distribution of the histopathological classification for the samples derived from the group with a positive PCR is presented in Table 3, while the detailed results for each patient are presented in Supplementary Material Table S2. Within this group of 44 patients, signs of bacterial colonization, inflammation, activity, atrophy, and metaplasia were observed among 39 (88.6%), 43 (97.7%), 24 (54.5%), 2 (4.5%), and 1 (2.2%) patient, respectively.

Only for one of the patients (patient no. 60, Supplementary Material Table S2), for who two biopsy samples were collected from a different localization for PCR testing, were the PCR results discordant, where one was positive, while the second produced a negative result.

Seven samples with discordant results between the histology investigation and PCR-based diagnostic kit, as presented in Table 1, were subjected to the “in house” version of real-time PCR for the detection of the *ureA* gene. Two of the mentioned samples were negative in PCR and simultaneously positive in the histopathological investigation (numbers 64 and 90, Supplementary Material, Table S2), while five were positive in PCR and simultaneously negative in the histopathological investigation (numbers 2, 9, 77, 91, and 104, Supplementary Material Table S2). The results of the *ureA* gene detection for each of the samples were concordant with the results of the applied commercially available test (Supplementary Material Tables S1 and S2) and the HRM specificity of the amplification is presented in Figures S1 and S2 in the Supplementary Material.

Additionally, DNA derived from two of these samples underwent DNA sequencing, and the *H. pylori* DNA presence was confirmed in both of them (samples from the patients numbers 2 and 9, Supplementary Material Table S1). Thus, the results of the *H. pylori* detection, at least for these samples, were confirmed with the application of three independent methods and were treated as truly-positive, despite having negative histopathological examination results.

## 3. Discussion

A number of methods are currently applied for the diagnosis of *H. pylori* infections in groups of pediatric patients [9,10,11,12]. For a reliable evaluation of the actual presence of bacteria in the gastric mucosa, invasive procedures for sample collection have to be applied. Thus, it is of great importance to choose tests that analyze the presence of bacteria at the site of infections and give reliable results, regardless of any influencing factors. Meanwhile, the sensitivity and specificity of *H. pylori* detection may vary significantly, depending on the investigation methodology, clinical specimen type, and treatment applied to the patient [13,14,15].

According to Zhang et al. (2018), no statistically significant differences have been noted in the detection rate of *H. pylori* strains with different genotypes, regardless of their eradication success. This underlines the usefulness of the PCR method for *H. pylori* detection among pediatric patients with respect to targeting particular genes [16].

Traditional methods for *H. pylori* detection, as it has been shown previously, may present low sensitivity, as bacteria growth *in vitro* is sometimes hard to reach. Therefore, a small number of bacteria may cause false-negative results in culture and histopathology investigation [17]. It has also been shown that the an urease breath test might give more positive results than the reference method [18]. Meanwhile, the results of Jeon et al. [19] showed a concordance between the results of the PCR and the conventional RUT in a number of samples, suggesting that the PCR-based test is a time- and cost-effective method for *H. pylori* detection.

Based on the results of this study, it can be concluded that among children, there is a high agreement between the results of *H. pylori* detection using histopathological investigation and molecular methods. Therefore, the reduction in the number of biopsies taken during endoscopy in children can be further discussed. As a consequence, this may affect the time and cost of the examination, especially histopathological testing, which is quite expensive, laborious, and time-consuming. If these observations could be confirmed on a larger group of children, perhaps it would be possible to reduce the cost and time of diagnostics in this way. Macroscopic assessment of the degree of inflammation, supported by molecular testing, would be a sufficient and reliable method for examining the changes induced by *H. pylori* and confirming colonization with this pathogen.

A good correlation between PCR and the histopathological investigation results was also confirmed previously by some researchers. For instance, using real-time PCR as a proposed new diagnostic standard, most cases were diagnosed correctly in the study conducted by Srebinska et al. [20]. The concordance of qPCR results and histology evaluation has been also confirmed for patients with minimal and atypical *H. pylori* infections [21,22]. Additionally, some authors claim that classical methods (UBT and histology) have a similar accuracy and might only need to be verified with PCR application in some relevant cases [23,24]. Therefore, it has recently been proposed to establish faster and more personalized approaches for the diagnosis of *H. pylori* infections [25,26].

In five cases presented in this study (patient nos. 2, 9, 77, 91, and 104, Supplementary Material Table S1), the results of the histopathological examination were negative, while the PCR produced positive results, which indicates the probable true positive results of the latter method. In four for these patients, inflammation was graded as 2 (moderate) on the Sydney scale and PPI treatment initiation was most likely to result in transient remission and clinical improvement. After discontinuation of the PPI therapy, there will most likely be a recurrence of symptoms. In such cases, the PCR method may be recommended if *H. pylori* was not detected in the histopathological examination, but a moderate or severe degree of inflammation was observed macroscopically.

It is definitely worth applying molecular testing more willingly than histopathological investigation in children during PPI treatment or shortly thereafter. In Poland, most PPIs are over-the-counter medicines and most patients suffer from abdominal pain; as a result, they introduce medications themselves or on the order of a doctor. This also happens before a gastroscopy, as the waiting time for a consultation with a gastroenterologist and referral for a gastroscopy is often long. The false-negative histopathological results noted in the present research might have been related to these situations.

Meanwhile, PCR-based methods showed the best performance for the detection of *H. pylori* in gastric samples. It has also been confirmed for patients suffering from achlorhydria, intestinal metaplasia, or gastric ulcer bleeding [27], regardless of previous PPI treatment, antibiotics or bismuth compound application. This diagnostic approach could become a new standard, especially in patients undergoing PPI treatment.

For the majority of the samples included in the study, both the PCR results and histological evaluation were concordant. For the PCR-negative samples, the compatibility of the histopathology investigation, confirmed by the lack of *H. pylori* colonization, reached 96.7% (58 out of 60 samples). Therefore, the sensitivity of the PCR-based methodology reached 95.3%, while its results specificity reached 92.6% when compared with the histopathology results. However, taking into account that two PCR-positive and histopathology-negative cases were also confirmed for *H. pylori* DNA, the calculated combined specificity and sensitivity parameters of the results should be significantly higher.

The corresponding value for the PCR- and histology-positive samples was 88.6% (39 out of 44 samples). Of note, among these five samples, the *ureA* gene was detected using the verification method. In addition, for two of these five discordant results, DNA sequencing was performed, which confirmed *H. pylori* DNA presence in the sample. Therefore, some of the authors underline that PCR-based examination is more sensitive than histopathology evaluation. Depending on the approach of the research, an exact PCR technique protocol can improve diagnosis by 11% compared with histopathological examination [28].

As it has recently been shown, PCR targeting the 16S rRNA-encoding gene shows the highest sensitivity with relatively high specificity results for the molecular detection of *H. pylori*, compared with the histopathological investigation results [21,22,29]. On the other hand, it has been shown that the selection of particular genes or sequences (namely, the gene for 16S rRNA) as molecular markers for *H. pylori* DNA detection [30] and as risk assessment of gastric cancers [31] are of great importance. A number of different PCR protocols have been introduced and analyzed in the studies of *H. pylori* infections diagnostics. They were conducted for the selection of optimal (conserved and reliable) genomic targets and primers/probes. This resulted in a different degree of specificity and sensitivity for the results of *H. pylori* DNA detection [32].

Usually, the 16S rRNA-encoding gene is chosen for the construction of bacterial diagnostic kits. However, for *H. pylori*, the *cagA*, *vacA*, *ureA*, and *glmM* genes are also of great importance. As it has been previously shown, a high specificity but low sensitivity, or the other way around, may be achieved using different sets of primers for *H. pylori* DNA detection [27,33,34]. It is always a matter of acceptance of the diagnostic methodology parameters. Regardless of the chosen gene, all of the commercially available kits underwent a detailed validation before their introduction for sale.

There was a statistically significant difference between the applied method and the obtained results, indicating that the PCR-based methodology might present higher diagnostic parameters than those of the histopathological investigations. Among the biopsy samples collected for the current research from 104 children, 44 (42.3%) were positive for the PCR investigation and mostly presented signs of inflammation and *H. pylori* colonization in a histological investigation, namely 43 (41.3%) and 39 (37.5%), respectively. An average grade of histopathologically-confirmed changes in the group of PCR-positive biopsy samples reached 2.00 and 2.39 for the density of *H. pylori* colonization and inflammation signs, respectively. Thus, the PCR results are concordant with the histopathological investigation effects, and confirm the differences between the results noted for the PCR-negative samples and the corresponding values that reached 0.03 and 0.80. The remaining average grades values for the other histopathological changes were also higher exclusively in the PCR-positive samples.

PCR-based diagnostics using non-invasive specimens on pediatric patients (stool and saliva) also deserve a brief discussion. These types of specimens usually need some additional preparation procedures for efficient DNA extraction. Nevertheless, taking into account the accessibility of the samples, it is worth considering them as specimens for a routine *H. pylori* detection [9,10,12,14]. However, the studies by some researchers show that the reliability of *H. pylori* DNA detection results performed for stool or saliva samples might be lower in terms of their sensitivity or specificity [13,35].

As it has been show previously, the severity of gastric mucosal damage is correlated with the presence of mutations in the gastric mucous cells and the age of patients [36]. Therefore, it may also influence the reliability of the PCR-investigated samples derived from the older patients. Based on the results of our own study, the histopathological changes might be correlated with the positive PCR results in a group of pediatric patients. Thus, molecular methods targeting a specific DNA sequence of the 16S rRNA gene might be relatively sensitive for the detection of *H. pylori* DNA. The primers/probes applied in the tested kit fortunately omit the potential mutations in the conserved bacterial DNA sequences, resulting in a high specificity for the obtained results.

As there is no gold standard technique for the diagnostics of patients with culture-negative results, there is a constant need to combine, compare, and correlate different laboratory investigation methods results. Altogether, they may help with a reliable diagnosis and prognosis of patients, especially for *H. pylori>* detection when invasive procedures for sample collection are already involved [37].

It is commonly known that the results of molecular testing depend mostly on the particular techniques applied or the type of clinical specimen used [13,15,38,39,40,41,42]. The usefulness of molecular methods for *H. pylori* infection diagnosis in children has been previously studied. The same issues were noted for real-time PCR [35,43] and also for some particular commercially available tests. However, the PCR diagnostic accuracy depends on different aspects. One of the most important features is the choice of a specific targeted DNA sequence [44]. Therefore, for the reliable detection of *H. pylori*, it would be advantageous to use a PCR reaction targeting different genes simultaneously.

As one of the PCR negative samples was positive in both histology and RUT, the PCR result was found to be a false-negative. Nevertheless, further studies are necessary to confirm this observation. Undoubtedly, the exact place for specimen collection is of the greatest relevance for the reliability of the results. In the present study, for one of the patients, two biopsy samples (antrum and stomach body) were collected for PCR testing, of which one was positive and the second was negative. It may also explain the differences in the results of the applied methods.

The present study has some limitations resulting from the study scheme design and accessibility of the samples. The most important are as follows: RUT was performed only for a limited group of samples; only one commercially available test based on PCR was applied for *H. pylori* detection (however supported with the *ureA* gene “in house” real-time PCR test as a verification method for the discordant results); and due to failure of *H. pylori* culture, the classical technique was discontinued at some research point. Because the biopsy culture was not performed for all of the samples, it was finally excluded from the results section.

In the present research, two PCR-negative samples observed among the samples with histopathological changes might be due to other bacterial infection, such as *Helicobacter* non-*Helicobacter pylori,* which also cause inflammation or other pathological symptoms [45,46]. This would explain the fact that in the present research among a group of PCR-negative samples, the highest value for histopathological changes was observed in the inflammation grade investigation. Interestingly, both of the mentioned samples were negative for the *H. pylori*-specific *ureA* gene in a verification investigation. This observation needs further studies to reveal the actual status of these samples in a group of pediatric patients. It could confirm the specificity of the results obtained with both applied methods, preferably when performed on more numerous clinical samples. Nevertheless, considering that PCR is a very specific and sensitive method and, additionally, it is less time-consuming and costly, it is worth discussing this method as a new standard in the diagnosis of *H. pylori* infection, at least among pediatric patients, taking into account the fact that a number of samples might be considered culture-negative or while inflammation signs are observed macroscopically and histopathological examination is negative.

## 4. Materials and Methods

### 4.1. Sample Collection and Histopathological Data

The research was carried out on samples (gastric biopsies) collected from 104 patients (58 females and 46 males) of the Department of Pediatric Endoscopy and Gastrointestinal Function Testing of Ludwik Rydygier Collegium Medicum in Bydgoszcz, Nicolaus Copernicus University in Toruń, Poland. The range of the patients’ age was 2 years 10 months to 17 years 11 months (median ± SD, 13 ± 4 years).

The sampling criteria were: chronic or recurrent not-functional abdominal pain (102 patients) or severe bleeding of the upper gastrointestinal tract (2 patients). Five samples were collected twice from the corresponding five patients for whom the efficacy of antimicrobial therapy was analyzed in the follow-up study or the initial results were ambiguous (patient nos. 58, 59, 60, 72, and 96).

Four biopsy samples (two from the stomach antrum and two from the stomach body) were collected from each patient and subjected to histopathology examination (0–3 graded evaluation in the modified Sydney classification), while an additional one was subjected for DNA isolation followed by *H. pylori* DNA detection with molecular methods. Five additional samples were obtained from five patients in the follow-up antimicrobial treatment effectiveness analysis (all dedicated for PCR testing exclusively).

Samples collected from 22 patients (Supplementary Material Tables S1 and S2) with the most visible and distinct morphological changes observed during gastroscopy (hallmarks seen macroscopically) were additionally used for biochemical RUT (Gold Hp dry, Lencomm, Santiago, Chile), performed according to manufacturer’s instructions.

All of the biopsy samples, directly after the collection steps, were preserved in a transport medium (BD BBL™ Port-A-Cul™ transport systems, Becton Dickinson, Franklin Lakes, NJ, USA) and immediately transported either for histopathological investigation or for DNA isolation purposes (one morphologically changed antrum sample per patient).

Each sample was histologically checked for the presence of particular pathological changes under the microscope using hematoxylin–eosin staining. The following determinants were investigated using the modified Sydney classification: (i) presence/density of *H. pylori* colonization, (ii) inflammation (infiltrates of lymphocytes and plasmocytes in the mucosa), (iii) inflammation activity (neutrophilic activation/infiltrates), (iv) glandular atrophy, and (v) intestinal metaplasia. All of the parameters were expressed in the Sydney classification scale at 0–1–2–3 grades for the advancement of the observed changes. The micrographs presenting examples of the histopathological investigation are presented as Supplementary Material, Figures S3–S7.

Based on the results of the histopathological investigation, the average grades of the changes in each parameter were calculated for the whole group of patients.

### 4.2. Culture of a Reference Strain

*H. pylori* reference strain (DSMZ 21031 purchased from Deutsche Sammlung von Mikroorganismen und Zellkulturen, Germany, https://www.dsmz.de/collection/catalogue/details/culture/DSM-21031, accessed on 14 December 2022) was used for the DNA isolation and as a positive control for all molecular-based methods. This type of strain was isolated from a gastric cancer patient in Australia and a full genome sequence is available. The culture of the reference strain was successfully achieved on selective media specific for *H. pylori* (Gelose Pylori agar, *bio*Mérieux) in microaerophilic conditions (Genbox Microaer, *bio*Mérieux, Marcy-l’Étoile, France) at 37 °C after 72 h of incubation.

### 4.3. DNA Isolation

The biopsy samples for DNA isolation were initially homogenized mechanically for approximately one minute. This was done through the application of manual homogenizers (Squisher-Single, ZymoResearch, Irvine, CA, USA) that fit to 1.5 mL Eppendorf-like test tubes. The samples, vigorously crushed and homogenized, were then subjected to 30 min digestion in 200 microliters of trypsin solution (5 mg/mL, Trypsin EDTA solution, Sigma, Darmstadt, Germany) at 37 °C to facilitate DNA extraction efficiency. Altogether, the DNA was extracted for 109 biopsy samples derived from 104 patients (Supplementary Material Tables S1 and S2), with the use of GeneProof Pathogen Free DNA Isolation Kit (GeneProof, Brno, Czech Republic) specific for clinical samples and performed according to the manufacturer’s recommendations. The DNA samples were then frozen at −20 °C before further investigation.

### 4.4. H. pylori DNA Detection

The AmpliSens^®^ *Helicobacter pylori*-FRT PCR Kit (AmpliSens^®^, Bratislava, Slovak Republic) based on real-time PCR for 16S rDNA was conducted according to the manufacturer’s recommendations, on a cobas z480 device (Roche, Basel, Switzerland). The results of the *H. pylori* DNA presence, expressed as C_T_, were set using the histological examination results for each patient (Supplementary Material, Table S1 for PCR-positive samples), while the remaining results are shown in Supplementary Material Table S2 (PCR-negative samples).

To check the reproducibility of the PCR results, each individual DNA sample was subjected to PCR two or three times.

The DNA extracted from the reference strain was used as a positive control for the results, while the molecular biology grade water (EurX, Gdańsk, Poland) served as a negative control of amplification.

### 4.5. Verification Methods—ureA Gene Detection and DNA Sequencing

Additional experiments were performed to verify the presence of *H. pylori* DNA in seven investigated samples (sample nos. 2, 9, 64, 77, 90, 91, and 104, Supplementary Material Tables S1 and S2). Real-time PCR for the *ureA* gene detection and high-resolution melt cure genotyping analysis were applied for this purpose. The mentioned methodology was applied for seven samples with discordant results between the histology investigation and the PCR-based diagnostic kit results. The following primers for the *ureA* gene amplification were used: AGTTCCTGGTGAGTTGTTCTT and TGGAAGTGTGAGCCGATTT, according to the study by Hasyanee Binmaeil et al. [47]. The presence of the *ureA* gene was determined using the real-time PCR method in the CFX Opus 96 Real-Time PCR Instrument (Bio-Rad, Feldkirchen, Germany). Positive (DNA extracted form *H. pylori* reference strain DSMZ 21031) and negative controls (molecular biology grade sterile water) were used simultaneously. The reactions were performed with the application of molecular biology grade sterile water (EurX, Gdańsk, Poland), primers (Genomed, Poznań, Poland), and the 5x HOT FIREPol^®^ EvaGreen^®^ HRM Mix (no ROX) reaction mixture (Solis BioDyne, Tartu, Estonia). The reaction volume for one sample consisted of 20 µL with the following: 4 µL of HRM Mix, both primers used at the final concentration of 200 nM, water (5 µL), and DNA template (1 µL). The amplification program consisted of the following: initial denaturation at 95 °C for 3 min, followed by 50 cycles of amplification, each consisting of 10 s at 95 °C and 20 s at 60 °C, and a final step at 72 °C for 20 s. After the amplification reaction, the high resolution melting (HRM) curves protocol was applied (95 °C for 5 s, 65 °C for 60 s and constant heating until reaching 97 °C with ramp rate 0.11 °C/s and five read-outs per °C) for the confirmation of the real-time PCR products’ specificity (Supplementary Material, Figures S1 and S2).

The DNA derived from two samples (patients 2 and 9—Supplementary Table S1) with discordant results for the histopathological investigation and PCR underwent DNA sequencing. Sequencing of the bacterial 16S rRNA-encoding DNA was carried out using the NGS method, through sequencing by synthesis, according to the methodology described by Salamon et al. [48]. The 10 pM indexed amplicons were pooled and mixed with 30% spike-in PhiX control DNA and then all loaded onto the MiSeq (Illumina, San Diego, CA, USA) apparatus according to Salamon et al. [48]. Sequencing was performed using the MiSeq Reagent Kit v3 (600 cycles).

### 4.6. Statistical Analysis

The two-tailed Fisher’s exact test was used to evaluate the statistically significant differences between the results of the histopathological evaluation and PCR testing (α ≤ 0.05 considered statistically significant).

The diagnostic parameters of the PCR results (sensitivity and specificity) were also calculated with respect to the histopathological results (treated as the reference method) according to a commonly known formula. The sensitivity was calculated as the percentage value of true positive results divided by the sum of the true positive and false negative results. The specificity was calculated as the percentage value of the true negative results divided by the sum of the true negative and false positive results.

## 5. Conclusions

PCR-based methods are of great importance for a reliable and fast diagnosis of *H. pylori* infections among pediatric patients. The results of the PCR are mostly in accordance with the histopathological investigation of the samples collected from the patients with *H. pylori* infections. Taking into consideration that PCR-based methods are less time-consuming and costly, and their results remain reliable, it is worth discussing these methodologies as a new standard in the diagnosis of *H. pylori* infections. It should be considered at least among pediatric patients, for which there is a high risk of a failure in the bacterial culture as well as discrepancies between the histopathological investigation and inflammation signs observed macroscopically.

## Figures and Tables

**Table 1:** Comparison of the results obtained with application of PCR with respect to their histological investigation results (*n* = 104).

Method/Result		PCR Result		Number (%) of Samples
		Positive	Negative	
*H. pylori* colonization in histology evaluation	positive	39 (37.5%)	2 (1.9%)	41 (39.4%)
	negative	5 (4.8%)	58 (55.8%)	63 (60.6%)
Total		44 (42.3%)	60 (57.7%)	104 (100%)

**Table 2:** Distribution of the histopathological investigation grades (modified Sydney classification) observed in the samples derived from the group of patients with negative PCR results (*n* = 60).

Histopathology results (modified Sydney classification)	**Colonization ** **Density Grade**	***n***	**(%)**	**Inflammation** **Grade**	***n***	**(%)**	**Inflammation ** **Activity/Atrophy/** **Metaplasia Grade**	***n***	**(%)**
	1	2	3.3	2	8	13.3	0	60	100.0
	0	6	10.0						
	0	32	53.3	1	32	53.3			
	0	20	33.3	0	20	33.3			
Average grade in a group	0.03			0.80			0.00		

**Table 3:** The detailed distribution of histopathological investigation grades (modified Sydney classification) observed in the group of patients with positive PCR results (*n* = 44).

Histopathology results (modified Sydney classification) for *H. pylori* infection identification	**Colonization Density Grade**	***n***	**(%)**	**Inflammation ** **Grade**	***n***	**(%)**	**Inflammation Activity Grade**	**Atrophy Grade**	**Metaplasia Grade**	***n***	**(%)**
	3	19	43.2	3	12	27.3	2	0	0	5	11.4
							1	0	1	1	2.3
							1	0	0	4	9.1
							0	0	0	2	4.5
				2	6	13.7	2	0	0	1	2.3
							1	0	0	3	6.8
							0	0	0	2	4.5
				1	1	2.3	0	0	0	1	2.3
	2	11	25.0	3	7	15.9	2	2	0	1	2.3
							2	0	0	3	6.8
							1	0	0	2	4.5
							0	0	0	1	2.3
				2	4	9.1	1	1	0	1	2.3
							1	0	0	1	2.3
							0	0	0	2	4.5
	1	9	20.4	3	3	6.8	2	0	0	1	2.3
							0	0	0	2	4.5
				2	4	9.1	1	0	0	1	2.3
							0	0	0	3	6.8
				1	2	4.5	0	0	0	2	4.5
	0	5	11.4	2	4	9.1	0	0	0	4	9.1
				0	1	2.3	0	0	0	1	2.3
Average grade in a group	2.00			2.39			0.80	0.07	0.02		

## Data Availability

The data presented in this study are available upon request from the corresponding author.

## Supplementary Materials

The following are available online at https://www.mdpi.com/article/10.3390/ijms24010179/s1.
