# Supplementary material for: Usefulness of Molecular Methods for Helicobacter pylori Detection in Pediatric Patients and Their Correlation with Histopathological Sydney Classification
Source: Int J Mol Sci. 2022 Dec 22;24(1):179. doi: 10.3390/ijms24010179 (PMC9820059; doi:10.3390/ijms24010179)
Supplement: Supplementary file 1 [file ijms-24-00179-s001.zip › ijms-2017350-supplementary.pdf]

Communication

Usefulness of Molecular Methods for *Helicobacter pylori* Detection in Pediatric Patients and Their Correlation with Histopathological Sydney Classification

Tomasz Bogiel [1,2,*], Agnieszka Mikucka [1,2], Anna Szaflarska-Popławska [3] and Dariusz Grzanka [4]

[1] Department of Microbiology, Ludwik Rydygier Collegium Medicum in Bydgoszcz, Nicolaus Copernicus University, 85-094 Bydgoszcz, Poland
[2] Clinical Microbiology Laboratory, Dr Antoni Jurasz University Hospital No. 1, 85-094 Bydgoszcz, Poland
[3] Department of Pediatric Endoscopy and Gastrointestinal Function Testing, Ludwik Rydygier Collegium Medicum in Bydgoszcz, Nicolaus Copernicus University, 85-094 Bydgoszcz, Poland
[4] Department of Clinical Pathomorphology, Faculty of Medicine, Collegium Medicum in Bydgoszcz, Nicolaus Copernicus University, 85-094 Bydgoszcz, Poland
* Correspondence: t.bogiel@cm.umk.pl; Tel.: +48-52-585-44-80

Table S1. The detailed results of histopathological investigation, rapid urea test (if performed), and cut off (C_T) values of real-time PCR investigation for PCR-positive samples (n = 44)

Patient/ sample No.	C_T of the first PCR	Rapid urease test result	Colonization density grade	Inflammation grade	Inflammation activity grade	Atrophy grade	Metaplasia grade
97	17.19	N/A	3	3	2	0	0
29	17.51	negative	3	2	1	0	0
105	17.91	positive	3	2	2	0	0
101	18.05	N/A	2	3	2	0	0
103	18.54	positive	1	2	0	0	0
98	18.57	N/A	3	3	1	0	0
27	18.86	N/A	1	1	0	0	0
102	18.93	N/A	3	3	2	0	0
4	19.47	positive	3	3	1	0	1
41	20.45	N/A	2	2	0	0	0
42	20.47	N/A	2	2	1	0	0
11	20.74	N/A	1	1	0	0	0
44	20.87	N/A	2	3	0	0	0
99	21.02	N/A	3	3	2	0	0
96	21.96	N/A	1	2	0	0	0
91*	22.26	N/A	0	2	0	0	0
5	22.45	N/A	2	3	2	0	0
3	22.52	positive	2	2	0	0	0

73	22.54	positive	3	3	2	0	0
32	22.7	N/A	3	3	0	0	0
9*,**	22.7	N/A	0	2	0	0	0
40	22.77	N/A	1	3	0	0	0
45	22.92	N/A	1	3	0	0	0
74	22.95	positive	2	3	2	0	0
1	23.04	N/A	3	2	0	0	0
79	23.47	positive	3	2	1	0	0
83	23.5	positive	3	3	2	0	0
2*,**	24.3	positive	0	2	0	0	0
82	24.47	positive	2	3	2	2	0
81	25.01	N/A	2	2	1	1	0
100	25.43	N/A	1	3	2	0	0
86	25.44	N/A	2	3	1	0	0
48	25.65	N/A	2	3	1	0	0
59	25.84	N/A	3	3	0	0	0
80	26.52	positive	3	3	1	0	0
84	26.65	N/A	3	3	1	0	0
58	26.73	N/A	3	3	1	0	0
87	27.04	N/A	3	2	1	0	0
13	28.21	N/A	3	1	0	0	0
78	28.3	N/A	3	2	0	0	0
76	30.99	negative	1	2	1	0	0
77*	31.98	N/A	0	2	0	0	0
31	32.21	N/A	1	2	0	0	0
104*	33.2	negative	0	0	0	0	0

N/A – the data not applicable; * - positive results obtained also for *ureA* gene detection in the "in house" testing based on real-time PCR; ** - presence of DNA for *H. pylori* 16S RNA genes was confirmed with DNA sequencing (sequencing of DNA coding bacterial 16S rRNA carried out using the NGS method—sequencing by synthesis. The 10 pM indexed amplicons were pooled and mixed with 30% spike-in PhiX control DNA and next all was loaded onto the MiSeq (Illumina) apparatus according to Salamon et al. (https://doi.org/10.1007/s00253-022-12251-z). Sequencing was performed using the MiSeq Reagent Kit v3 (600 cycles).

Table S2. The detailed results of histopathological investigation for PCR-negative samples (n = 60)

Patient/ sample No.	Rapid urease test result	Colonization density grade	Inflammation grade	Inflammation activity grade	Atrophy grade	Metaplasia grade
6	N/A	0	0	0	0	0
7	N/A	0	1	0	0	0
8	N/A	0	0	0	0	0
10	N/A	0	0	0	0	0
12	N/A	0	1	0	0	0
15	N/A	0	0	0	0	0
16	N/A	0	1	0	0	0
17	N/A	0	0	0	0	0
18	negative	0	1	0	0	0
19	N/A	0	1	0	0	0
20	N/A	0	0	0	0	0
21	N/A	0	0	0	0	0
22	N/A	0	1	0	0	0
23	N/A	0	1	0	0	0
24	N/A	0	0	0	0	0
25	N/A	0	1	0	0	0
26	N/A	0	1	0	0	0
28	negative	0	1	0	0	0
30	N/A	0	1	0	0	0
33	N/A	0	1	0	0	0
34	N/A	0	0	0	0	0
35	N/A	0	1	0	0	0
36	N/A	0	1	0	0	0
37	N/A	0	1	0	0	0
38	N/A	0	0	0	0	0
39	N/A	0	0	0	0	0
43	N/A	0	0	0	0	0
46	N/A	0	2	0	0	0
47	N/A	0	2	0	0	0
49	N/A	0	1	0	0	0
50	N/A	0	0	0	0	0
51	N/A	**0**	0	0	0	0
52	N/A	0	2	0	0	0
53	N/A	0	0	0	0	0

54	negative	0	1	0	0	0
55	N/A	0	0	0	0	0
56	N/A	0	2	0	0	0
57	N/A	0	1	0	0	0
60	N/A	0	1	0	0	0
61	N/A	0	2	0	0	0
62	N/A	0	2	0	0	0
63	N/A	0	1	0	0	0
64*	positive	1	2	0	0	0
65	negative	0	1	0	0	0
66	negative	0	1	0	0	0
67	N/A	0	0	0	0	0
68	N/A	0	1	0	0	0
69	N/A	0	0	0	0	0
70	N/A	0	1	0	0	0
71	N/A	0	1	0	0	0
72	N/A	0	2	1	0	0
75	negative	0	1	0	0	0
85	N/A	0	1	0	0	0
88	N/A	0	1	0	0	0
89	N/A	0	0	0	0	0
90*	N/A	1	2	0	0	0
92	positive	0	1	0	0	0
93	N/A	0	1	0	0	0
94	N/A	0	1	0	0	0
95	N/A	0	1	0	0	0

N/A – the data not applicable; * - negative results obtained also for *ureA* gene detection in the "in house" testing based on real-time PCR

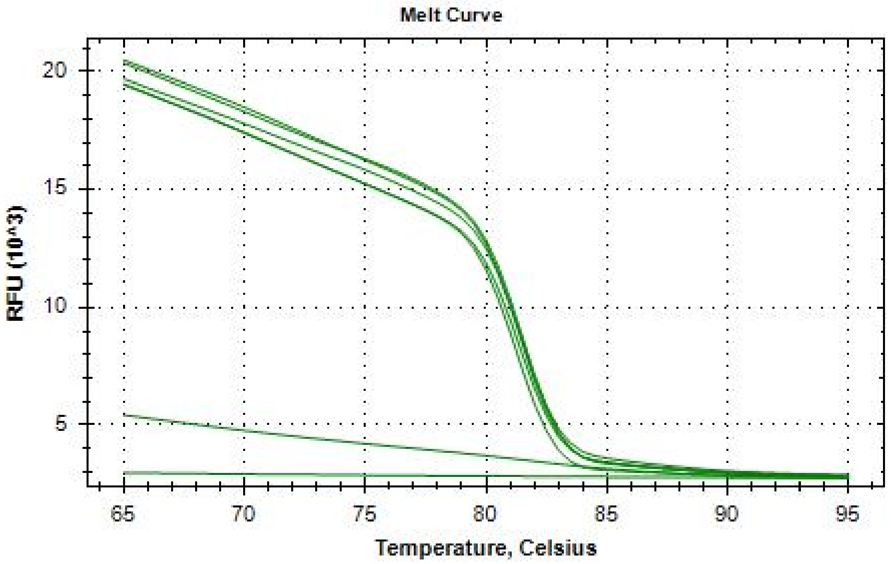

Figure S1. Melt curves for particular samples; investigation using High Resolution Melting technique showing specificity of "in house" real-time PCR for *ureA* gene

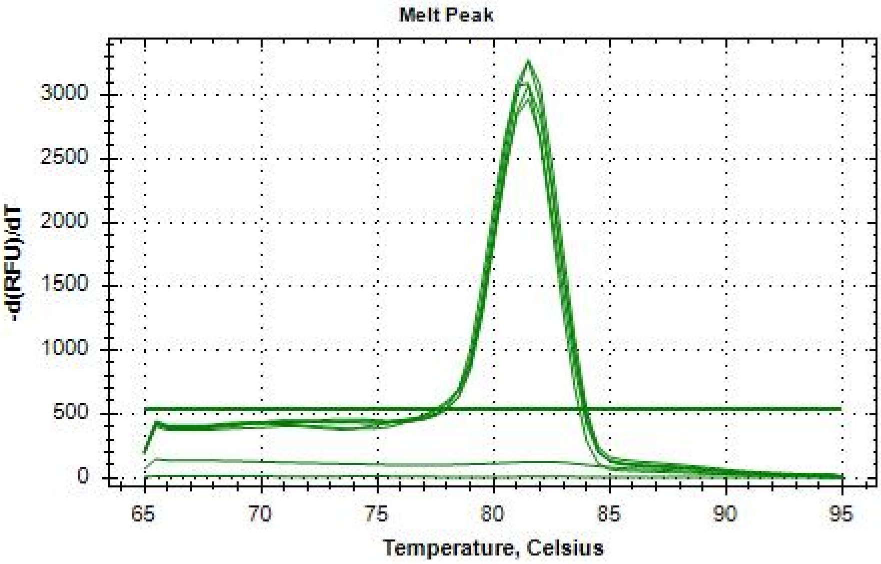

Figure S2. Melt peaks for particular samples; investigation using High Resolution Melting technique showing specificity of "in house"real-time PCR for *ureA* gene

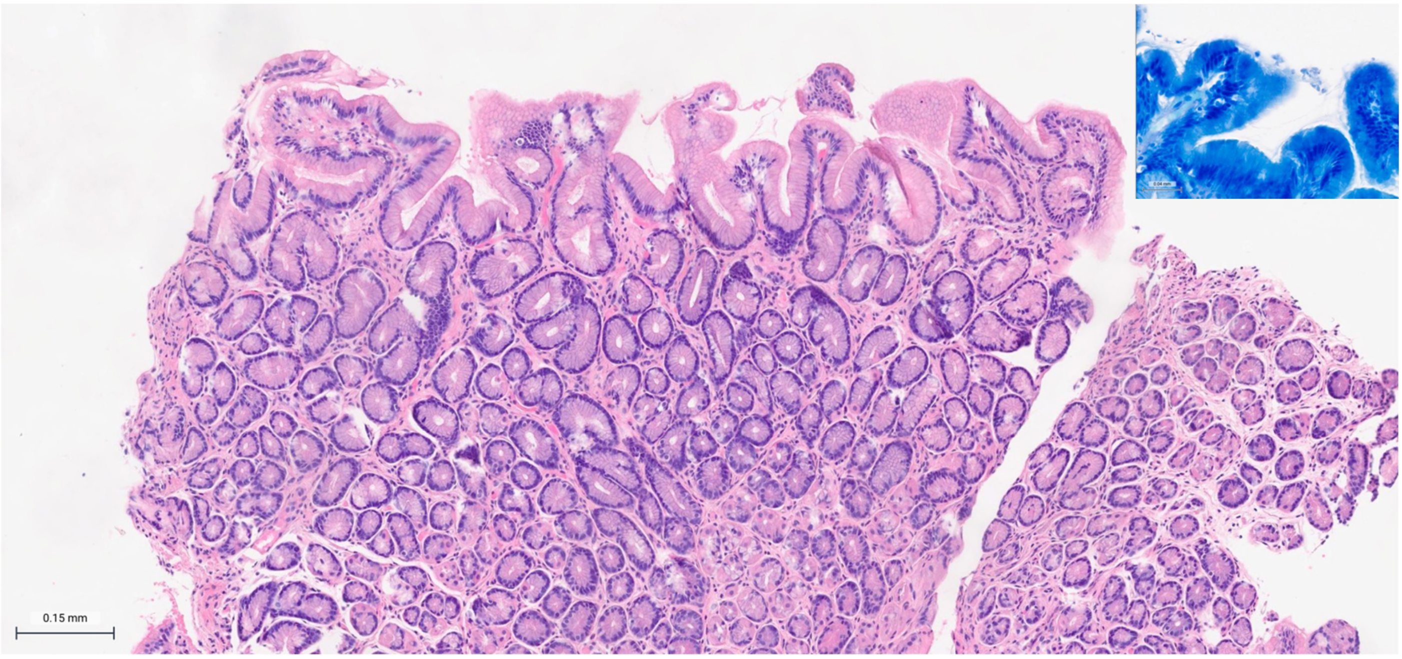

Figure S3. The sample derived from the patient No. 104, histopathologically normal, *H. pylori* DNA detected with PCR, hematoxylin-eosin staining, 10x magnification (basic micrograph) and Giemsa staining

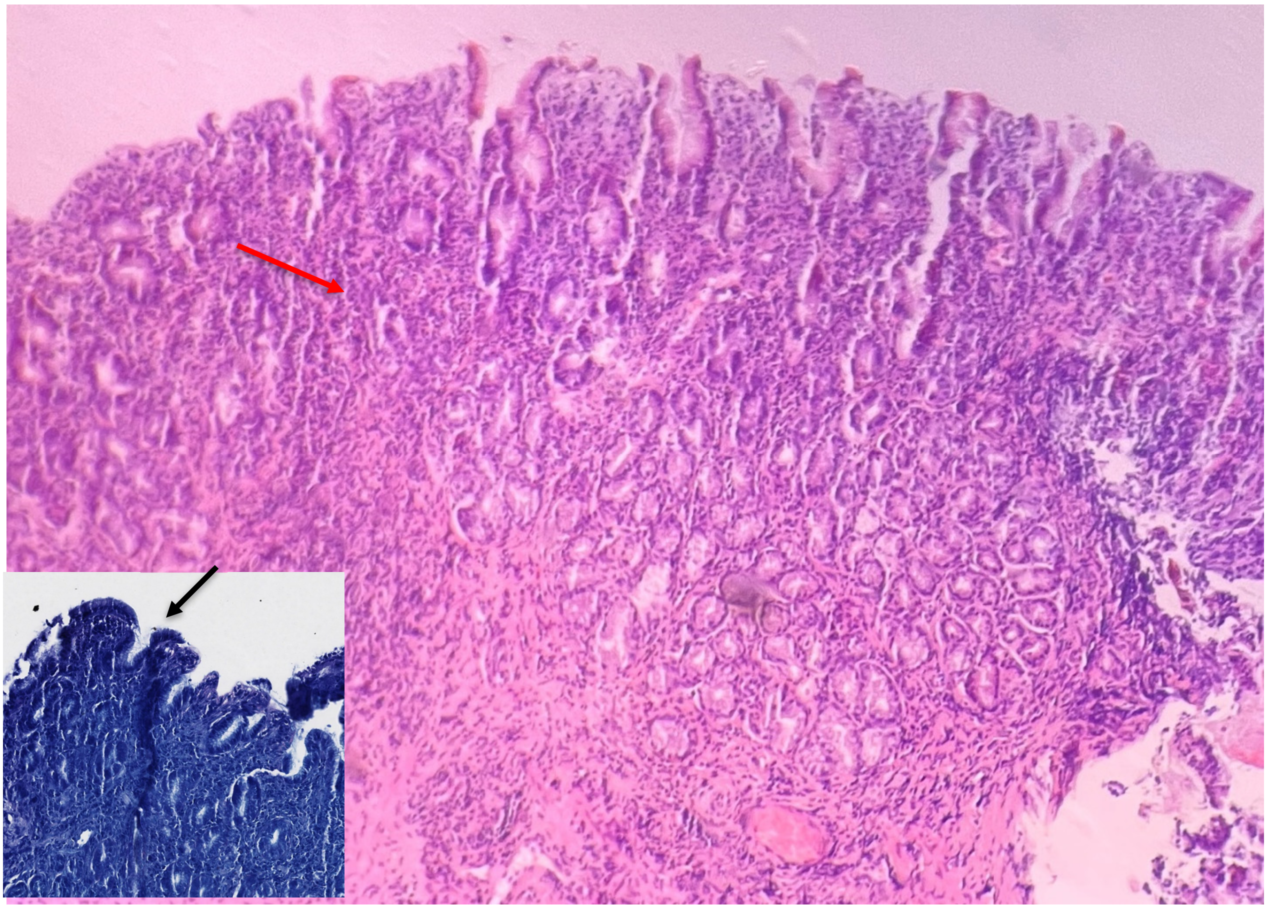

Figure S4. The sample derived from the patient No. 27, *Helicobacter pylori* colonization density (**black arrow**) and inflammation grade 1 (**red arrow**), hematoxylin-eosin staining, 10x magnification (basic micrograph) and Giemsa staining

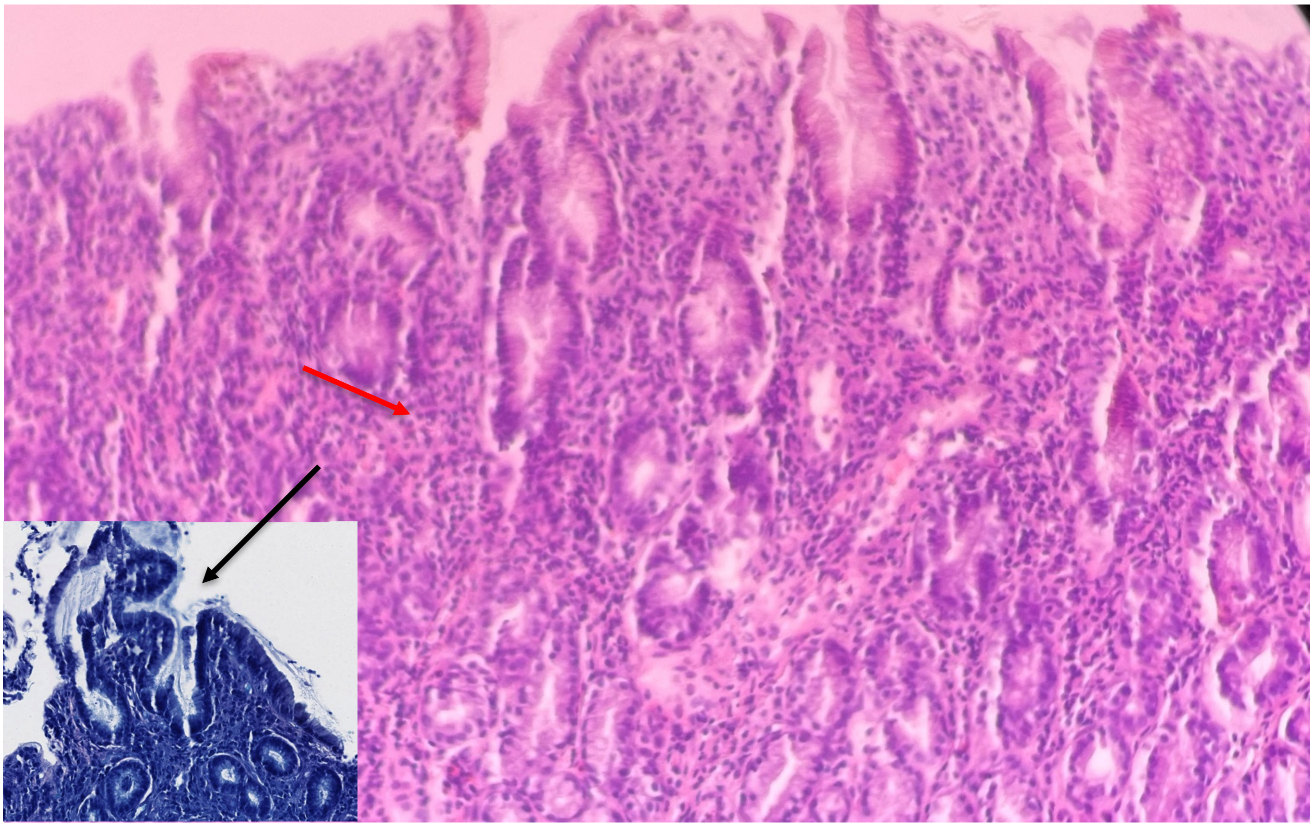

Figure S5. The sample derived from patient No. 27, *Helicobacter pylori* colonization density (**black arrow**) and inflammation grade 1 (**red arrow**), hematoxylin-eosin staining, 20x magnification (basic micrograph) and Giemsa staining

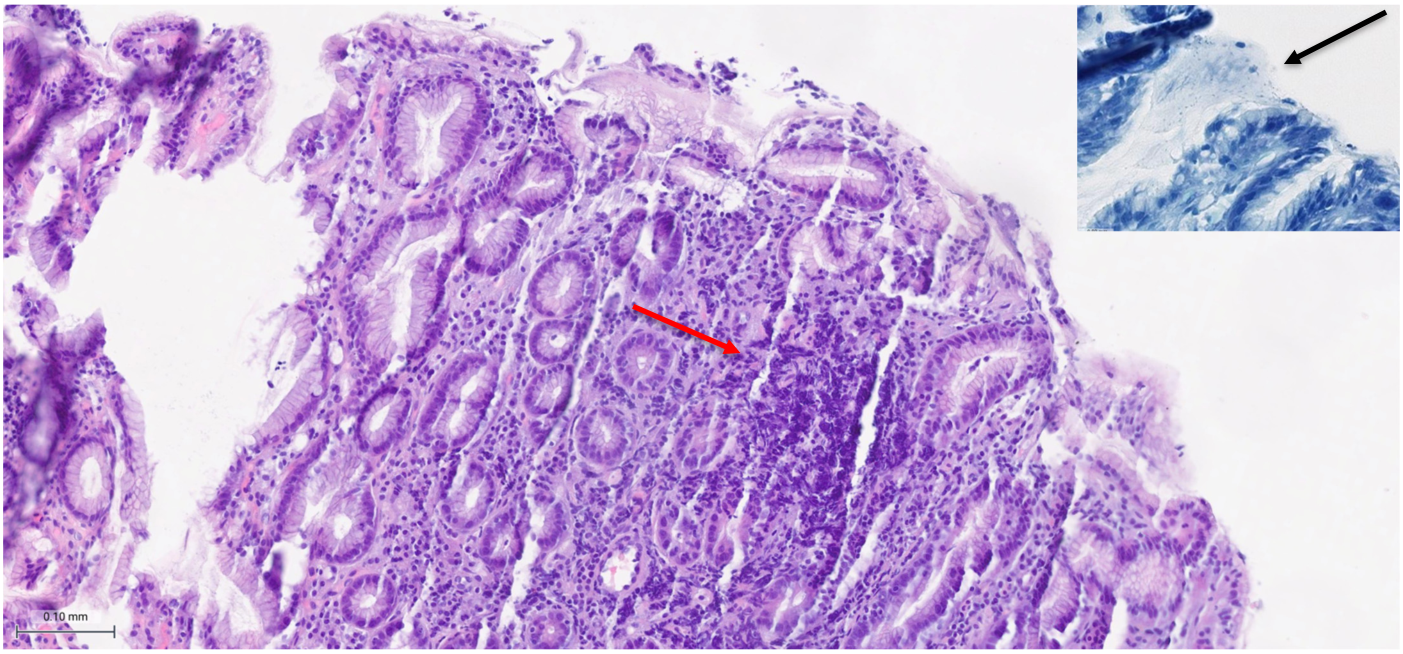

Figure S6. The sample derived from the patient No. 82, *Helicobacter pylori* colonization density (**black arrow**), inflammation activity, and atrophy grade 2, inflammation grade 3 (**red arrow**), hematoxylin-eosin staining, 10x magnification (basic micrograph) and Giemsa staining

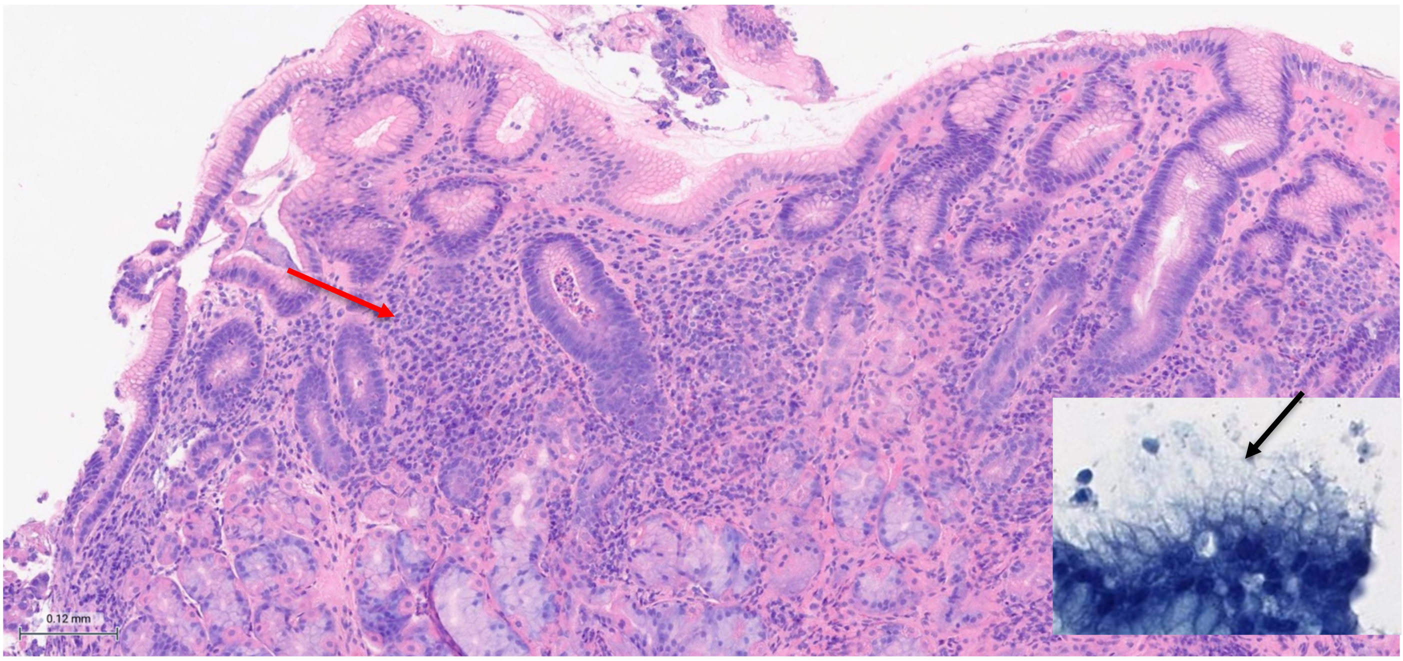

Figure S7. The sample derived from the patient No. 105,
Helicobacter pylori colonization density grade 3 (**black arrow**), inflammation and inflammation activity grade 2 (**red arrow**), hematoxylin-eosin staining, 10x magnification (basic micrograph) and Giemsa staining
